# FSA food allergen alerts: an analysis of trends in reporting

**DOI:** 10.1186/2045-7022-5-S3-P125

**Published:** 2015-03-30

**Authors:** Katie Waters, Rahul Chodhari

**Affiliations:** 1 Watford General Hospital, London, United Kingdom; 2 Royal Free Hospital, London, United Kingdom

## Background

Up to 10% of the paediatric population is affected by food allergy. Affected families face the treat of acute life-threatening reactions, and the burden of chronic decrease in quality of life. UK legislation exists where allergens must be indicated on food labels; any inaccuracies identified must be rapidly revised, and the public informed. This is achieved through Food Standards Agency (FSA) allergy alerts which can be viewed on their website; consumers may also sign up to receive emails or SMS messages when alerts are issued.

## Aim

The aim of this study is to inform health professionals of trends food allergy reporting and to assess the accessibility of this information for parents, in order to provide opportunities to improve allergen alert reporting.

## Methods

Individual allergy alerts published on the FSA website from 2011 to 2013 were analysed, alongside additional data obtained from annual reports dating back to 2008. Further information was gained through discussion with parents regarding their engagement with the FSA website.

## Results

**Figure 1:**
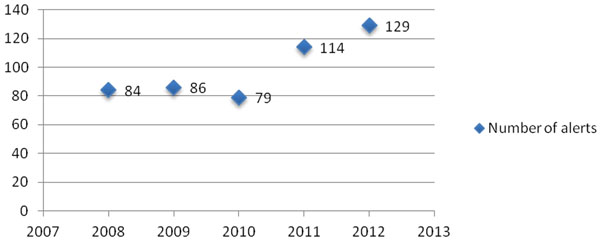
FSA reported allergy alerts

**Figure 2:**
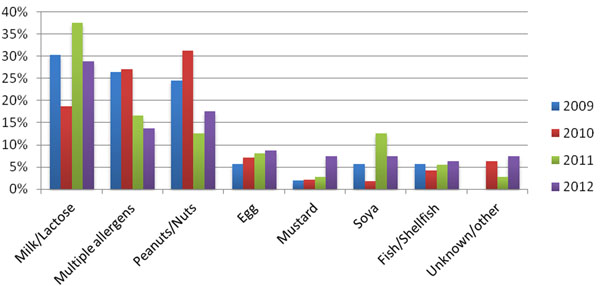
Allergens reported in food alerts

The results of the study revealed the following:

• Year-on-year increase in reporting of alerts: 53.4% increase from 2008-2012 (Fig 1).

• Large supermarkets previously reported the majority of allergen alerts (64% in 2011) but increasing number of reports from small businesses mean the proportion of reports from supermarkets has now decreased (52% in 2013).

• The majority of alerts involve milk and nuts, accounting for 43% of reports in 2012 (Fig 2).

• Main contaminated food groups triggering alerts are nut products, ready meals, chocolate and sweet products.

• Alerts were found most commonly to result from labelling errors.

• 100% of small business and supermarkets recall or withdraw their product once an alert is identified, but supermarkets were found to be more rigorous in taking further measures to inform and refund customers.

• Informal discussion with parents of children attending allergy clinics revealed that parents are not aware of the information available on the FSA website.

## Conclusion

Food allergy alerts (mainly involving milk and nuts) are increasing, yet it seems consumers are not accessing these reports. The main source of errors is incorrect labelling, most commonly involving ready meals and chocolate/sweet products. Dissemination of these findings to retailers may result in error reduction. Informing parents of allergy alerts may increase allergen avoidance and improve confidence in allergen reporting, increasing quality of life.

